# Adherence to Heart-Healthy Behaviors in a Sample of the U.S. Population

**Published:** 2005-03-15

**Authors:** Chris L Bryson, Rosalie R Miller, Anne E Sales, Branko Kopjar, Stephan D Fihn

**Affiliations:** Health Services Research and Development, Northwest Center of Excellence, VA Puget Sound Health Care System; The author is also affiliated with the Department of Medicine, University of Washington, Seattle, Wash; Health Services Research and Development Center of Excellence, VA Puget Sound Health Care System, Seattle, Wash; Centers for Disease Control and Prevention, Division of Nutrition and Physical Activity, Atlanta, Ga; Health Services Research and Development Center of Excellence, VA Puget Sound Health Care System, Seattle, Wash, and Department of Health Services, University of Washington, Seattle, Wash; Health Services Research and Development Center of Excellence, VA Puget Sound Health Care System, Seattle, Wash, and Department of Health Services and Department of Medicine, University of Washington, Seattle, Wash

## Abstract

**Introduction:**

Following national recommendations for physical activity, diet, and nonsmoking can reduce both incident and recurrent coronary heart disease. Prevalence data about combinations of behaviors are lacking. This study describes the prevalence of full adherence to national recommendations for physical activity, fruit and vegetable consumption, and nonsmoking among individuals with and without coronary heart disease and examines characteristics associated with full adherence.

**Methods:**

We performed a cross-sectional analysis of data from the 2000 Behavioral Risk Factor Surveillance System, a national population-based survey. We included respondents to the cardiovascular disease module and excluded individuals with poor physical health or activity limitations.

**Results:**

Subjects were most adherent to smoking recommendations (approximately 80%) and less adherent to fruit and vegetable consumption and physical activity (approximately 20% for both). Only 5% of those without coronary heart disease and 7% of those with coronary heart disease were adherent to all three behaviors (*P* < .01). Among those without a history of coronary heart disease, female sex (odds ratio [OR] 1.47; 95% confidence interval [CI], 1.23–1.76), highest age quintile (OR 1.67; 95% CI, 1.28–2.19), more education (OR 2.48; 95% CI, 1.69–3.64), and more income (OR 1.19; 95% CI, 1.04–1.36) were associated with full adherence. Among those with coronary heart disease, mid-age quintile (OR 3.79; 95% CI, 1.35–10.68), good general health (OR 2.05; 95% CI, 1.07–3.94), and more income (OR 1.51; 95% CI, 1.06–2.16) were associated with full adherence.

**Conclusion:**

These data demonstrate the lack of a heart-healthy lifestyle among a sample of U.S. adults with and without coronary heart disease. Full adherence to combined behaviors is far below adherence to any of the individual behaviors.

## Introduction

Physical inactivity, unhealthy diet, and smoking are three leading health behaviors contributing to the prevalence of coronary heart disease (CHD) in the United States (1,2). Significant research effort goes into documenting the prevalence of these behaviors and quantifying how much they contribute to CHD morbidity and mortality. While prevalence and risk data are available for individual behaviors, data about combinations of behaviors are lacking.

National recommendations for physical activity, diet, and smoking abstinence are available for all Americans. For most individuals, even those with CHD, walking is practicable. And for all individuals, independent of CHD or health status, maintaining a healthful diet and smoking cessation are achievable goals. The intent of these recommendations is different for those without and for those with CHD, namely to prevent incident cases of heart disease in those at risk and to reduce the risk of subsequent events in those with prevalent disease. While smoking is clearly associated with heart disease, a growing body of literature demonstrates that exercise, including moderate activity such as walking (3,4), and increased fruit and vegetable intake reduce the risk of CHD events (5-7). There are currently some data on the level of adherence to individual health behaviors.

Approximately one in five of the U.S. population adheres to recommendations for fruit and vegetable intake (8), one in four adhere to recommendations for exercise (9), and three in four adheres to recommendations not to smoke (10). Yet adherence to these individual recommendations is still below national goals (2), and there are very limited data on the combinations of these behaviors.

For public health, combinations of risk factors and behaviors are at least as important to understand as the prevalence of single behaviors. Major risk factors, including cholesterol, hypertension, and smoking clearly are responsible for the vast majority of heart disease (11,12). Furthermore, recommendations for diet, exercise, and smoking abstinence impact these major cardiovascular risk factors in different ways. Those with a low-risk profile have a much lower incidence of cardiovascular disease (CVD) than those with one or more single risk factors (13), and the lowest incidence of heart disease appears to be among those who adhere to multiple risk-reducing behaviors (14). The prevalence of individuals with multiple favorable risk factors or who participate in multiple positive health behaviors is known to be relatively rare (12-14). A full picture of modifiable cardiovascular risk cannot be accurately reflected in separate estimates of adherence to single behaviors.

We have sought 1) to describe the prevalence of full adherence to national recommendations for physical activity, fruit and vegetable consumption, and smoking abstinence, and 2) to examine characteristics associated with full adherence in a sample of the U.S. population, stratified by CHD status.

## Methods

### Design

We used the 2000 Behavioral Risk Factor Surveillance System (BRFSS) survey to provide the data for our study. The  BRFSS is a population-based, random-digit–dialed telephone survey of the civilian, noninstitutionalized U.S. population aged 18 years and older. The methodology of BRFSS is described in detail elsewhere (15). The following summary focuses on our analysis of respondents with and without self-reported CHD.

### Study population

Thirteen states (Delaware, Georgia, Indiana, Iowa, Kentucky, Mississippi, Montana, Ohio, Oklahoma, Pennsylvania, South Carolina, Virginia, and West Virginia) and the District of Columbia collected information about the prevalence of CVD in 2000. We selected participants who answered the CVD module (module 13) and then divided this group into individuals with and individuals without self-reported heart disease based on the following questions: 1) Has a doctor ever told you that you had a heart attack or myocardial infarction? 2) Has a doctor ever told you that you had angina or coronary heart disease? Individuals who responded yes to questions 1 or 2 were defined as having CHD.

We excluded individuals who reported poor physical health or activity limitation for at least a period of 15 days in the preceding month in both individuals with and individuals without CHD. These individuals would not be expected to participate regularly in exercise because of poor health and are therefore not included in the denominator of people who could potentially participate in these recommendations. Subjects were asked two questions to quantitate the number of days that they had poor physical health: 1) Now thinking about your physical health, which includes physical illness and injury, how many days during the past 30 days was your physical health not good?; 2) During the past 30 days, for about how many days did poor physical or mental health keep you from doing your usual activities, such as self-care, work, or recreation?  Individuals who had 15 or more days of poor health from either question were excluded.

Responses of "don't know/not sure" to any of the questions or refusals to give an answer were coded as missing for this analysis.

### Ascertaining adherence status

For those individuals with and individuals without self-reported heart disease, we extracted data on the following three health behaviors: physical activity (core question 6), smoking status (core question 7), and fruit and vegetable intake (core question 8) (Appendix). These behaviors were divided into dichotomous categories classified as fully adherent or not fully adherent to national recommendations.

### Physical activity

Respondents were asked about the two physical activities that they engage in most often and about the frequency and duration of each activity. Respondents were classified as fully adherent if they engaged in a moderate-intensity physical activity at least five days per week for at least 30 minutes per day or vigorous-intensity activity at least three days per week for at least 20 minutes per day during the preceding month, based on recommendations from the Centers for Disease Control and Prevention (CDC) and the American College of Sports Medicine (16). We defined activity on the basis of metabolic equivalent (MET) intensity levels recorded in the compendium of physical activities: light activity, <3 METs; moderate activity, 3–6 METs; and vigorous activity, >6 METs (16). One MET is defined as the energy expended by sitting quietly and is equivalent to 3.5 ml of O_2_ uptake per kg body weight per minute, or to 1 kcal/kg of body weight per hour. Using the list of 56 recorded activities in the BRFSS and each activity's associated MET, we computed the self-reported frequency and duration of each activity per individual for both moderate-intensity and vigorous-intensity categories. Respondents were included if they had complete data for either the primary or secondary activity. If the primary or secondary activity met recommendations, or if the summation of primary and secondary activities met recommendations, respondents were counted as fully adherent. Responses with incomplete information about frequency or duration of activity could not be evaluated for adherence and were coded as missing.

### Smoking abstinence

Respondents were asked if they had smoked 100 cigarettes in their entire lifetime and to categorize their current smoking pattern. Current nonsmokers (never and former smokers) were classified as fully adherent, without regard to the length of time since ceasing to smoke.

### Fruit and vegetable intake

Respondents were classified as fully adherent to fruit and vegetable intake recommendations if they reported consuming five or more servings per day of combined fruits, fruit juice, and vegetables based on recommendations from the U.S. Department of Health and Human Services (17) and the American Heart Association (18). Participants were asked to report their consumption frequency of five foods: fruit, fruit juice (such as orange, grapefruit, or tomato), green salad, carrots, and other vegetables. Each participant was asked how frequently he or she consumed an item. Because the BRFSS did not inquire about portion size, each affirmative response was counted as one portion. Responses with insufficient data to determine the servings per day of a group were coded as missing for that group. Participants with data for at least four out of the five groups were included for the analysis. The groups were added together to produce the total number of fruit and vegetable servings per day. Participants reporting a total of five or more servings per day were counted as fully adherent to dietary recommendations for fruit and vegetable consumption.

We excluded potatoes because a growing body of evidence shows that a high intake of starchy vegetables, including potatoes, does not provide protective benefits against CHD and may increase risk of CHD (6,19,20). While the benefit of fruit juice on incident CHD risk has not been clearly established, fruit juice has not been found to confer an increased risk of CHD and was counted towards the fruit and vegetable total servings.

### Combined adherence to physical activity, nonsmoking, and diet

A composite, dichotomous adherence score was calculated for each individual, incorporating physical activity, diet, and nonsmoking data. Respondents were classified as fully adherent to all three behaviors or as not fully adherent. Subjects were included only if they had enough information to count them as either fully adherent or not fully adherent to each individual behavior. A total of 1594 records (4%) were missing full adherence data (diet, 749; physical activity, 746; and current smoking status, 99) and were excluded from the analyses.

### Statistical analysis

We computed age- and sex-adjusted prevalence estimates by direct standardization, using the entire group of respondents to the CVD module as the referent population. Broad age categories were used in the adjustment, as attempts to refine the age categories further produced inaccurate results because of the small numbers of fully adherent respondents. Statistical significance was tested using an age- and sex-adjusted logistic regression that accounted for the study design, modeling the characteristic as either a dichotomous or an indicator variable. Because those with CHD comprised 7% of the total population, and the rate of full adherence was low, race and body mass index (BMI) categories were collapsed to produce column proportions. All variables were modeled as categorical indicator variables unless noted.

Logistic regression was used to examine characteristics associated with full adherence, while adjusting for covariates. Multivariate models included the following covariates: age (18–99, in quintiles), gender, race (white, nonwhite), income (<$15,000; $15,000–$24,999; $25,000–$49,999; ⩾$50,000), education (<high school, high school or GED, >high school), general health (good, not good), mental health (good, not good), and BMI (underweight and normal, <18.5 kg/m^2^ and 18.5–24.9 kg/m^2^; overweight, 25–29.9 kg/m^2^; and obese, ⩾30 kg/m^2^). Age was divided into quintiles to provide sufficient numbers for analysis. Indicator variables were used for age, education, and BMI to model possible non-linear effects. Income was included as a grouped linear variable. Because the multivariable models revealed no difference in the odds of adherence for respondents with greater than or equal to high school education among those with CHD, the two higher educational categories were combined to create a dichotomous variable (<high school, ⩾high school) among those with CHD. Sociodemographic and health status characteristics were chosen a priori. Multivariable modeling with numerous covariates was not possible given the smaller number of respondents within the CHD group and the low prevalence of full adherence overall. Efforts were made to keep models comparable for both those with and those without CHD. All variables were entered into the model simultaneously.

All *P* values, adjusted odds ratios, and confidence intervals were obtained from logistic regression analyses that accounted for sampling design using Stata 7 SE (Stata Corp, College Station, Tex). All *P* values are two-tailed.

## Results

Of the 43,058 individuals who answered both the CHD questions and the exercise, fruit and vegetable, and smoking questions, 3167 (7.4%) reported CHD. Our final sample included 38,851 subjects. The final sample excluded individuals who were likely to be unable to comply with physical activity recommendations; they reported more than 15 days of limitation because of poor health or they reported physical health as not good for more than 15 days. Among the final sample, 2189 (5.6%) reported CHD. As expected, there were disproportionately more subjects with self-reported CHD who were excluded for 15 or more days of poor health (n = 978, 30.9% of those with CHD) compared with those without CHD (n = 3229, 8.1% of those without CHD). Overall, only 5.1% of those without CHD and 7.2% of those with CHD fully adhered to all three health behaviors (unadjusted *P* value for difference = .009). When adjusted for age and sex, 5.0% of those without CHD and 5.1% of those with CHD were fully adherent (*P* = .03 for difference).

We examined how combinations of adherence to individual recommendations overlapped and used Venn diagrams to illustrate the frequency of the patterns of overlap. Figures 1a and 1b describe the weighted, unadjusted proportion of individuals adhering to seven possible individual and combined behaviors and the proportion of individuals who did not adhere to any of the behaviors. Regardless of CHD status, a minority of individuals were fully adherent to all three recommended behaviors. Adherence to single recommendations and to combinations of recommendations was similar in both groups. Adherence to fruit and vegetable intake and smoking abstinence was higher among individuals with CHD (*P* = .007, fruit and vegetable intake; *P* = .008, smoking abstinence), while there was no significant difference between CHD and non-CHD groups in the proportion of individuals who exercise (*P* = .10). Most respondents who were adherent to physical activity recommendations were also nonsmokers. Most respondents who adhered to fruit and vegetable intake recommendations were also nonsmokers. However, among individuals with and individuals without CHD, nearly one half of individuals who were adherent to nonsmoking recommendations did not adhere to either of the other two health behaviors. The proportion of individuals who did not follow any of the recommendations was moderate and similar for both those with CHD (16%) and those without CHD (18%).

**Figure 1a:**
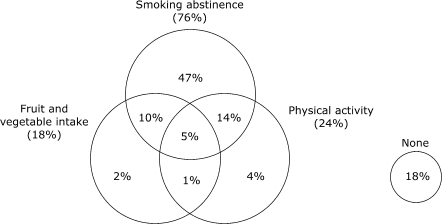
Proportion of respondents without heart disease adherent to individual and combinations of fruit and vegetable intake, nonsmoking, and physical activity recommendations (n, sample = 36,772; N, weighted population = 40,725,302).

**Table:** A text description of this diagram is also available

**Figure 1b:**
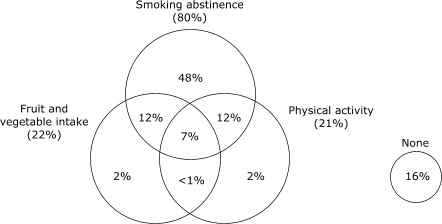
Proportion of respondents with heart disease adherent to individual and combinations of fruit and vegetable intake, nonsmoking, and physical activity recommendations (n, sample = 2129; N, weighted population = 2,361,137).

**Table:** A text description of this diagram is also available

Tables 1 and 2 show the characteristics of fully adherent individuals (column proportions labeled Full Adherence) and the age- and sex-adjusted proportions of individuals adherent to single recommendations and the combination of the three among specific groups (row proportions labeled Smoking Abstinence, Physical Activity, Fruit and Vegetable Intake, and All). The adjusted proportion of individuals who were fully adherent by individual characteristics varied from 2% to 7% among individuals without CHD (Table 1) and from 1% to 9% among those with CHD (Table 2). Among those without CHD, the higher proportion of adherence observed for women was associated with a greater adherence to fruit and vegetable intake but relatively similar levels of adherence for the other two behaviors. The lower adherence observed for the youngest age group was associated with a combination of lower adherence to smoking and diet recommendations, despite having better adherence to physical activity. Higher levels of income and education were associated with greater adherence to all three behaviors. Similar trends were seen for income and education among those with CHD.

We also examined characteristics independently associated with full adherence using logistic regression (Table 3). Among individuals without a history of CHD, highest age quintile [61–99 years] (OR 1.67; 95% CI, 1.28–2.19), female sex (OR 1.47; 95% CI, 1.23–1.76), nonwhite race (OR 1.29; 95% CI, 1.03–1.63), more education (OR 2.48; 95% CI, 1.69–3.64), more income (OR 1.19; 95% CI, 1.04–1.36), good general health (OR 1.39; 95% CI, 0.96–1.20) and good mental health (OR 1.50; 95%CI, 0.96–2.32) were associated with full adherence. In those with CHD, age quintile 3 [63–69 years] (OR 3.79; 95% CI, 1.35–10.68), age quintile 4 [70–76 years] (OR 3.42; 95% CI, 1.20–9.76), more income (OR 1.51; 95% CI, 1.06–2.16), and good general health (OR 2.05; 95% CI, 1.07–3.94) were associated with full adherence.

## Discussion

We evaluated full adherence to three heart-healthy behaviors in a sample of individuals from 13 states and the District of Columbia with and without CHD. We found prevalence estimates for adherence to each single behavior that were similar to other studies (8,21-24). Approximately 18% of those without CHD and 22% of those with CHD were adherent to fruit and vegetable intake recommendations. Li et al report similar rates of adherence of 19%, 22%, and 23% for BRFSS respondents in the years 1990, 1994, and 1996 (8). We also found approximately 76% of those without CHD and 80% of those with CHD were current nonsmokers, similar to a national sample from the 2001 National Health Interview Survey that found approximately 77% of adults overall were nonsmokers. We found that 21% of those with CHD and 24% of those without CHD were adherent to physical activity recommendations, also similar to prior published BRFSS data from 1998 in which 25% of the overall population participated in recommended levels of physical activity (22). Most strikingly, we also observed that only about one in 20 individuals was adherent to all three of these behaviors, far below adherence to any of the individual behaviors.

Similar to other work in health-related behavior (25,26), we found both an education gradient and income gradient, with prevalence for all three behaviors being highest among individuals with more than high school education and greatest income. Higher levels of education may increase the likelihood of obtaining or understanding health-related information needed to develop heart-healthy behaviors. And while recent health gains for the U.S. population as a whole appear to reflect achievements among higher socioeconomic groups, lower socioeconomic groups continue to lag (2).

While we found that most respondents classified as adherent to physical activity or fruit and vegetable intake recommendations were also nonsmokers, the lower full adherence observed for the youngest age group for those without CHD was associated with a combination of lower adherence to nonsmoking and fruit and vegetable intake, despite having better adherence to physical activity. Among those with CHD, the lower full adherence observed for the youngest age group was associated with a combination of lower adherence to all three behaviors, with smoking accounting for the largest absolute difference between the youngest and oldest groups.

Among those without CHD, we also identified greater full adherence for self-identified nonwhite, nonblack racial groups (i.e., "other"), which was largely related to better adherence to fruit and vegetable intake. It is possible that cultural influences on food preferences account for better adherence and would have been recognized had ethnicity, rather than racial categories, been reported.

Consistent with other reports, we found that full adherence to dietary recommendations varied by age and sex, with the better dietary habits reported in women among those without CHD and reported in older adults in both CHD groups (27). However, even among these groups, low adherence to individual recommendations was common, suggesting that there is substantial room for improvement. Full adherence to fruit and vegetable intake was comparable for men and women with CHD and likely represents the greater proportion of men in our sample.

Whether affecting the risk for a first cardiovascular event or recurrent event, studies conclusively show that engaging in lifestyle modification reduces the risk of future coronary events (3-5,28-32). Major risk factors explain the bulk of cases of heart disease, and these risk factors are directly related to diet, physical activity, and smoking (33). Hypertension, cholesterol levels, and smoking are the most potent risk factors for CVD (13,34,35). Smoking remains the leading preventable cause of death in the United States (34) and contributes substantially to cardiovascular mortality and morbidity. The relative risk of CHD associated with physical inactivity ranges from 1.5 to 2.4, an increase in risk comparable to that observed for high blood cholesterol, hypertension, or cigarette smoking (16). Multiple studies have reported inverse relationships between fruit and vegetable intake and risk of CVD (5,6,28). As previous work has shown, combinations of healthy behaviors have an incrementally more protective effect (14). Among a group of women without CVD at baseline, nonsmokers who maintained healthful diet and who followed physical activity recommendations had an incidence of coronary events that was 60% lower than the incidence among the rest of the population. Physical activity and healthy diet have been shown to be independently associated with reductions in CHD risk. These findings strongly argue for monitoring the prevalence of not only individual health behaviors in the general population but also the combination of adherence to diet, exercise, and smoking recommendations.

Health promotion and disease prevention programs aimed at CHD might be more effective if they aimed at particular segments of the population who have more than one behavioral risk factor for CHD. For example, if a large proportion of the population is sedentary and has unhealthy diets but does not smoke, then a two-pronged program that aims at improving diet and physical activity but spends little or no time on smoking abstinence could be considered.

This study had limitations. The BRFSS does not reach individuals without telephones. Telephone coverage has been found to be lower among poorer households, so lower income respondents in both CHD groups may have been underrepresented in BRFSS samples (36). The BRFSS is based on self-reported data and is subject to reporting error. Misclassification within our CHD groups may have occurred because of reporting error in the form of incomplete medical knowledge or reporting bias by the respondent. Because of the social desirability of these behaviors, this study may overestimate the number of people adherent to each recommendation and also combinations of behaviors. We also may have misclassified some individuals based on fruit and vegetable intake because of missing data; however, analyses including subjects with complete data did not differ from analyses including subjects with four out of the five fruit and vegetable responses.

The weighted findings provide prevalence estimates that apply to the 13 states and the District of Columbia that used the BRFSS CVD module in 2000, and there is no guarantee that those 13 states were representative of all 50. However, we have no reason to suspect that these states are dissimilar to the remainder of the states and territories that participate in the BRFSS survey. The response rate for the 2000 BRFSS was approximately 50%. Although some attempt is made through post-stratification reweighting to force the age, sex, and race composition of the weighted BRFSS population to agree with census totals, it is possible that respondents still differ from nonrespondents on other characteristics that may be relevant to CHD risk factors. We used current national guidelines to create definitions of adherence to fruit and vegetable intake, exercise, and smoking recommendations. These were based on the guidelines from the CDC and the American College of Sports Medicine (16) and the American Heart Association (37,38) for exercise; from numerous sources, including the American Heart Association, for not smoking (18,38); and from the U.S. Department of Health and Human Services (17) and the American Heart Association for fruit and vegetable intake (18). While there is no debate on appropriate smoking behavior for cardiovascular risk reduction and some debate on the type and amount of physical activity that is best for risk reduction, there is no single widely accepted consensus yet for the most appropriate dietary regimen. In examining only the amount of fruit and vegetable intake, we ignored other important dietary factors that are known to influence cardiovascular risk factors. These include other dietary patterns and nutrients, such as the Mediterranean diet, which is high in omega-3 fatty acids, and the DASH (Dietary Approaches to Stop Hypertension) diet, which is high in fruit servings but also very low in sodium. Nevertheless, generous fruit and vegetable intake has been associated with reductions in CVD and may reflect generally healthier diets. The consumption of at least five or more servings of fruit and vegetables is also endorsed by the American Heart Association Dietary Guidelines, in part because this dietary pattern generally represents a healthier eating pattern, and also data show reductions in cardiovascular risk associated with this type of eating pattern (18).

In summary, we found markedly low full adherence to combined national recommendations for physical activity, fruit and vegetable intake, and smoking abstinence in this population-based study among people with and people without CHD. Population-wide behavioral strategies that target combinations of unhealthy behaviors and that complement clinical strategies may be more effective in producing desired changes and reducing the risk of CVD, investments which are critically needed in reducing the nation's CHD burden.

## Figures and Tables

**Table 1:** Adjusted^a^ Prevalence Estimates of Full Adherence to National Recommendations on Smoking Abstinence, Physical Activity, and Fruit and Vegetable Intake for BRFSS^b^ Cardiovascular Disease Module Respondents Without Self-Reported Heart Disease

**(Sample, n = 36,722; Population, N = 40,725,302) **					
**Characteristic **	** Full Adherence^c^ (%) **	**Smoking Abstinence (%) **	**Physical Activity (%) **	**Fruit and Vegetable Intake (%) **	**All (%) **
**Sex **					

Female	61	77	22	22	6
Male	39	75	25	14	4

**Age, years**					

18-44	51	71	25	16	5
45-64	33	77	22	19	6
⩾65	16	90	21	22	6

**Race**					

White	83	75	24	18	5
Black	11	79	21	19	4
Other	6	78	23	25	6
**Married/partnered **	63	79	22	18	5

**Income**					

<$15,000	6	74	21	18	5
$15,000-$24,999	14	71	22	17	4
$25,000-$49,999	31	75	22	17	5
⩾$50,000	50	82	26	20	6

**Education**					

<High school	4	59	17	13	2
High school or GED	22	70	19	15	3
>High school	74	82	27	22	7

**Employment^d^**					

Employed	79	77	23	18	5
Retired	17	82	23	22	3
Unemployed	3	68	21	17	4
Unable to work	1	67	15	17	2

**General health^e^**					

Good	95	77	24	19	5
Fair	5	63	16	15	3
Poor	<1	59	13	16	3
**Good mental health^f^**	97	77	24	18	5

**BMI (kg/m^2^)**					

<18.5	2	73	20	19	6
18.5-24.9	50	73	27	19	6
25-29.9	37	78	23	18	5
⩾30	11	81	17	17	3
**Diabetes^g^**	5	81	19	22	6
**Stroke^h^**	1	70	21	15	6

a Characteristics are age and sex-adjusted, and percentages are weighted estimates.

b Abbreviations: BRFSS indicates Behavioral Risk Factor Surveillance System; GED indicates general equivalency diploma; BMI indicates body mass index.

c Percentages in the Full Adherence column reflect the proportion of subjects with the row characteristic who are adherent (row percentage), while the following four columns reflect the proportion of subjects who are adherent to smoking abstinence, physical activity, and fruit and vegetable recommendations among subjects defined by each row characteristic (column percentages).

d Employment categories are mutually exclusive; employed includes self-employed, employed by others, homemakers, and students.

e Subjects were asked, “Would you say in general your health is excellent, very good, good, fair, or poor?” Good health includes excellent, very good, and good responses.

f Good mental health is defined as ⩾15 days of self-reported good mental health in the preceding month.

g Subjects were asked, "Have you ever been told by a doctor that you have diabetes?"

h Subjects were asked, "Has a doctor ever told you that you had a [stroke]?"

**Table 2:** Adjusted^a^ Prevalence Estimates of Full Adherence to National Recommendations on Smoking Abstinence, Physical Activity, and Fruit and Vegetable Intake for BRFSS^b^ Cardiovascular Disease Module Respondents With Self-Reported Heart Disease

**(Sample, n = 2129; Population, N = 2,361,137) **					
**Characteristic **	**Full adherence^c^ (%) **	**Smoking Abstinence (%) **	**Physical Activity (%) **	**Fruit and Vegetable Intake (%) **	**All (%) **

**Sex **					

Female	31	69	15	19	4
Male	69	65	20	21	6

**Age, years **					

18-44	6	57	15	19	4
45-64	25	73	18	19	5
⩾65	68	89	22	24	9

**Race **					

White	88	66	17	20	5
Nonwhite	12	68	20	15	4
**Married/partnered **	64	68	17	18	5

**Income **					

<$15,000	3	67	10	22	3
$15,000-$24,999	11	64	15	20	3
$25,000-$49,999	25	70	13	20	4
⩾$50,000	61	69	18	25	8

**Education **					

<High school	9	52	11	13	4
High school or GED	20	65	17	17	3
>High school	71	71	20	24	7

**Employment^d^**					

Employed	76	68	17	18	5
Retired	20	64	11	10	4
Unemployed	3	29	12	21	9
Unable to work	1	70	14	35	1

**General health^e^**					

Good	82	67	20	19	6
Fair	14	61	12	20	3
Poor	4	82	6	35	2
**Good mental health^f^**	98	69	16	21	5

**BMI (kg/m^2^) **					

<24.9	45	64	23	18	6
25-29.9	40	63	20	22	4
⩾30	15	73	11	23	3
**Diabetes^g^**	24	70	12	29	6
**Stroke^h^**	9	69	22	25	5

a Characteristics are age and sex-adjusted, and percentages are weighted estimates.

b Abbreviations: BRFSS indicates Behavioral Risk Factor Surveillance System; GED indicates general equivalency diploma; BMI indicates body mass index.

c Percentages in the Full Adherence column reflect the proportion of subjects with the row characteristic among those who are adherent (row percentage), while the following four columns reflect the proportion of subjects who are adherent to smoking abstinence, physical activity, and fruit and vegetable recommendations, and the combination of all three among subjects defined by each row characteristic (column percentages).

d Employment categories are mutually exclusive; employed includes self-employed, employed by others, homemaker, and students.

e Subjects were asked, “Would you say in general your health is excellent, very good, good, fair, or poor?” Good health includes excellent, very good, and good responses.

f Good mental health is defined as ⩾15 days of self-reported good mental health in the preceding month.

g Subjects were asked, "Have you ever been told by a doctor that you have diabetes?"

h Subjects were asked, "Has a doctor ever told you that you had a [stroke]?"

**Table 3:** Characteristics Associated With Full Adherence to National Recommendations on Fruit and Vegetable Intake, Smoking, and Physical Activity Among BRFSS Cardiovascular Disease Module Respondents^a^

**Without CHD**		**With CHD**	

	**OR (95% CI) **		**OR (95% CI) **

**Age (years) **			

Quintile 1, (18-30)	1.00 (ref)	Quintile 1, (18-52)	1.00 (ref)
Quintile 2, (31-39)	0.92 (0.71-1.19)	Quintile 2, (53-62)	1.32 (0.47-3.72)
Quintile 3, (40-48)	0.98 (0.75-1.27)	Quintile 3, (63-69)	3.79 (1.35-10.68)
Quintile 4, (49-60)	1.26 (0.97-1.64)	Quintile 4, (70-76)	3.42 (1.20-9.76)
Quintile 5, (61-99)	1.67 (1.28-2.19)	Quintile 5, (77-97)	1.99 (0.54-7.29)

**Sex **			

Male	1.00 (ref)	Male	1.00 (ref)
Female	1.47 (1.23-1.76)	Female	1.23 (0.63-2.41)

**Race **			

White	1.00 (ref)	White	1.00 (ref)
Nonwhite	1.29 (1.03-1.63)	Nonwhite	0.83 (0.34-2.02)

**Education **			

<High school	1.00 (ref)	<High school	1.00 (ref)
High school or GED	1.21 (0.82-1.79)	⩾High school	1.24 (0.51-3.00)
⩾high school	2.48 (1.69-3.64)		

**Income **			

<$15,000	1.00 (ref)	<$15,000	1.00 (ref)
>$15,000	1.19 (1.04-1.36)	>$15,000	1.51 (1.06-2.16)

**General health**			

Fair or poor	1.00 (ref)	Fair or poor	1.00 (ref)
Good^b^	1.39 (0.96-1.20)	Good	2.05 (1.07-3.94)

**Mental health^c^**			

Poor	1.00 (ref)	Poor	1.00 (ref)
Good	1.50 (0.96-2.32)	Good	0.89 (0.22-3.65)

**Body Mass Index **			

⩽24.9	1.00 (ref)	⩽24.9	1.00 (ref)
25-29.9	0.81 (0.67-0.97)	25-29.9	0.52 (0.26-1.05)
⩾30	0.47 (0.37-0.60)	⩾30	0.88 (0.40-1.94)

a Abbreviations: BRFSS indicates Behavioral Risk Factor Surveillance System; CHD indicates coronary heart disease; OR indicates odds ratio; CI indicates confidence interval; GED indicates general equivalency diploma.

b Subjects were asked, “Would you say in general your health is excellent, very good, good, fair, or poor?” Good health includes excellent, very good, and good responses.

c Good mental health is defined as ⩾15 days of self-reported good mental health in the preceding month. Poor mental health is defined as <15 days of good mental health.

## Appendix. Behavioral Risk Factor Surveillance System Core Questions

### Section 6: Exercise

The next few questions are about exercise, recreation, or physical activities other than your regular job duties.

6.1. During the past month, did you participate in any physical activities or exercises such as running, calisthenics, golf, gardening, or walking for exercise?

6.2. What type of exercise or exercise did you spend the most time doing during the past month?

Ask Q6.3 only if answer to Q6.2 is running, jogging, walking, or swimming. All others, go to Q6.4.

6.3. How far did you usually walk/run/jog/swim?

6.4. How many times per week or per month did you take part in this activity during the past month?

6.5. And when you took part in this activity, for how many minutes or hours did you usually keep at it?

6.6. Was there another exercise or exercise that you participated in during the last month?    

6.7. What other type of exercise gave you the next most exercise during the past month?

Ask Q6.8 only if answer to Q6.7 is running, jogging, walking, or swimming. All others go to Q6.9.

6.8. How far did you usually walk/run/jog/swim?

6.9. How many times per week or per month did you take part in this activity?

6.10. And when you took part in this activity, for how many minutes or hours did you usually keep at it?

### Section 7: Tobacco Use

7.1. Have you smoked at least 100 cigarettes in your entire life?

7.2. Do you now smoke cigarettes every day, some days, or not at all?

7.3. On the average, about how many cigarettes a day do you now smoke?

7.3a. On the average, when you smoked during the past 30 days, about how many cigarettes did you smoke a day?

7.4. During the past 12 months, have you quit smoking for 1 day or longer?

7.5. About how long has it been since you last smoked cigarettes regularly, that is, daily?

### Section 8: Fruits and Vegetables

These next questions are about the foods you usually eat or drink. Please tell me how often you eat or drink each one, for example, twice a week, three times a month, and so forth. Remember, I am only interested in the foods you eat. Include all foods you eat, both at home and away from home.

8.1. How often do you drink fruit juices such as orange, grapefruit, or tomato?

8.2. Not counting juice, how often do you eat fruit?

8.3. How often do you eat green salad?

8.4. How often do you eat potatoes not including french fries, fried potatoes, or potato chips?

8.5. How often do you eat carrots?

8.6. Not counting carrots, potatoes, or salad, how many servings of vegetables do you usually eat?
